# Lyme disease: recent advances and perspectives

**DOI:** 10.3389/fcimb.2015.00027

**Published:** 2015-04-01

**Authors:** Tanja Petnicki-Ocwieja, Catherine A. Brissette

**Affiliations:** ^1^Division of Geographic Medicine and Infectious Diseases, Tufts Medical CenterBoston, MA, USA; ^2^Department of Basic Sciences, University of North DakotaGrand Forks, ND, USA

**Keywords:** Lyme Disease, *Borrelia burgdorferi*, *Ixodes scapularis*, innate immunity, Lyme arthritis, adhesins, c-di-GMP, Tnseq

Lyme Disease, caused by the spirochete *Borrelia burgdorferi* and transmitted by *Ixodes scapularis* (deer tick or blacklegged tick), has been gaining in incidence over the past decade. Without treatment, it is a long-term infection characterized by inflammation of the joints, heart and nervous system. The Centers for Disease Control and Prevention (CDC) classifies it as an Emerging Infectious Disease with an expanding geographical area of occurrence and have recently revised their incidence cases in the United States by 10 fold.

In this Lyme Disease Research Topic we have gathered reviews and original research in the fields of microbiology and immunology of *B. burgdorferi* infection. Included in this Topic are the abstracts from the 13th International Congress on Lyme Borreliosis and we thank the organizers Dr. Linda Bockenstedt and Dr. Linden Hu for publishing them in this issue at the following link http://www.frontiersin.org/books/13th_International_Conference_on_Lyme_Borreliosis_and_other_tick_Borne_Diseases_/357.

## Increased geographical distribution

As an emerging infectious disease the incidence and geographical distribution of Lyme disease cases are being monitored by a number of US states. Based on the CDC, Ohio is considered a non-endemic area for Lyme Disease, largely due to the low incidence of the arthropod vector *I. scapularis*. A tick surveillance program established by the Ohio Department of Public Health indicated a sharp increase in the prevalence of this tick in the state. Here, Wang et al. provide data that suggest an establishment of the enzootic cycle in Ohio (Wang et al., 2014).

## Vaccine development—a different approach

The development of a Lyme disease vaccine has been a hot topic for researchers and the public. Currently, the Food and Drug Administration has not given its approval for a human Lyme Disease vaccine. Therefore, researchers have sought alternate approaches to address control of *B. burgdorferi* infection in humans through pest management intervention. These methods have mainly consisted of vector-targeted or reservoir-targeted vaccines aiming to reduce tick density or control different aspects of the enzootic cycle. Gomes-Solecki writes a detailed review of the current status of those studies (Gomes-Solecki, 2014).

## Host immunity—novel pathways and genetic approaches

Because the majority of Lyme disease pathology is due to an over-exuberant immune response, much research in *B. burgdorferi* pathogenesis has been devoted to understanding the mammalian host response to the bacterium. A significant focus of immune studies has been the innate immune response as an initiator of inflammation. Recent studies have elucidated novel components of the innate immune response and intracellular pathways that participate in *B. burgdorferi* induced inflammation. Here, Cervantes et al. and Petnicki-Ocwieja and Kern review the most recent studies dissecting the numerous innate immune response pathways involved in *B. burgdorferi* recognition (Cervantes et al., 2014; Petnicki-Ocwieja and Kern, 2014).

Although much progress has been made in identifying immune pathways that participate in the *B. burgdorferi* induced response, it is unclear how these pathways lead to arthritis resistance or susceptibility. The field of Lyme disease has long debated the etiology of long-term inflammation and recent studies in the murine host have shed light on relevant cell types and inflammatory mediators that participate in the pathology of Lyme arthritis. Pratt and Brown review the role of eicosanoids as important mediators of arthritis (Pratt and Brown, 2014). Also, Bramwell et al. review the challenges of genome wide association studies for studying complex genetic traits in humans and the power of forward genetic approaches in model animals leading to the identification of genetic loci responsible for arthritis severity phenotypes (Bramwell et al., 2014). Finally, in a research paper, Belperron et al. present data that implicate the FcRY receptor as an important contributor to acute phase Lyme arthritis (Belperron et al., 2014).

## Lessons from the tick

Much of the research in Lyme Disease immunology has focused on understanding the immune response in the mammalian host. However, the importance of the enzootic cycle and the mode of survival of the bacterium in its arthropod host cannot be understated. The *I. scapularis* tick maintains the bacterium in its mid-gut, but precisely how tick immunity functions and influences persistence of pathogens remains unknown. Here, Smith and Pal discuss exciting insights that could be gained from mining the sequenced Ixodes genome and highlight future areas of investigation (Smith and Pal, 2014).

## What about the bug?

In nature, *B. burgdorferi* cycles between the vastly different environments of the *Ixodes* tick vector and mammalian host. *B. burgdorferi* must be able to detect changes in its environment, and rapidly respond to these changes. c-di-GMP, a second messenger unique to bacteria, is a global regulator that facilitates adaption to changing environmental circumstances. Novak et al. provide up-to-date information on how c-di-GMP signaling is instrumental in orchestrating the adaptation of *B. burgdorferi* to the tick environment (Novak et al., 2014).

Adhesion is the initial event in the establishment of any infection. *B. burgdorferi* modulates adhesion to host tissues in order to colonize, disseminate, and persist in its mammalian host. Brissette and Gaultney update current knowledge on the structure, function, and role of borrelial adhesins in Lyme disease pathogenesis (Brissette and Gaultney, 2014).

## The next generation: massively parallel sequencing tnseq

*B. burgdorferi* lacks many traditional virulence factors, such as toxins or specialized secretion systems. Therefore, studies focusing on host-pathogen interactions have been limited by an incomplete understanding of the repertoire of bacterial virulence factors. Questions such as how the pathogen causes disease, colonizes the tick and evades host immune-surveillance have been difficult to address.

Genetic studies involving single gene deletions have identified a number of important bacterial proteins, but a large-scale genomics approach to identify virulence factors has not been attempted until recently. Lin et al. review the generation of a site-directed mutagenesis library as an important step toward a detailed analysis of the *B. burgdorferi* genome and pathogenome (Lin et al., 2014). Using this library, high-throughput genomic studies, utilizing techniques such as massively parallel sequencing or Tnseq have shown to be a powerful tool in understanding the pathogen.

Lyme disease is endemic through much of the Northern hemisphere, including North America, Europe, and Asia, and will continue to be a public health concern for the foreseeable future. This Topic highlights the important work that is currently being done to understand the pathogen, the vector, and the disease. We hope that you find this Topic as enlightening and thought-provoking as we did.

### Conflict of interest statement

The authors declare that the research was conducted in the absence of any commercial or financial relationships that could be construed as a potential conflict of interest.
